# Severe neurological complications in critically ill COVID-19 patients

**DOI:** 10.1007/s00415-020-10152-7

**Published:** 2020-08-14

**Authors:** Quirin Notz, Christopher Lotz, Johannes Herrmann, Marius Vogt, Tobias Schlesinger, Markus Kredel, Wolfgang Muellges, Dirk Weismann, Thomas Westermaier, Patrick Meybohm, Peter Kranke

**Affiliations:** 1 Department of Anesthesiology and Critical Care, University Hospital Wuerzburg, Wuerzburg, Germany; 2 Department of Diagnostic and Interventional Neuroradiology, University Hospital Wuerzburg, Wuerzburg, Germany; 3 Department of Neurology, University Hospital Wuerzburg, Wuerzburg, Germany; 4 Department of Internal Medicine I, University Hospital Wuerzburg, Wuerzburg, Germany; 5 Department of Neurosurgery, University Hospital Wuerzburg, Wuerzburg, Germany

Dear Sirs,

A 56-year-old male patient was admitted to a secondary care hospital 7 days after the onset of fever and coughing. COVID-19 was confirmed and the patient was intubated after rapid respiratory deterioration. He was transferred to our tertiary care center and admitted to the intensive care unit (ICU). The patient suffered from a severe acute respiratory distress syndrome (ARDS, p_a_O_2_/F_i_O_2_ 64 mmHg [15], Fig. 1c, d), septic shock [17] and acute renal failure. He received low-dose acetylsalicylic acid (ASA) and therapeutic anticoagulation with unfractionated heparin (activated partial thromboplastin time, aPTT, in the range of 43–76 s). Over the next few days, the pulmonary gas exchange and other organ functions ameliorated. On day 18, however, routine clinical examination revealed a unilateral, dilated and unresponsive pupil, which lead to an immediate cranial computed tomography (cCT). The cCT showed excessive bilateral parieto-occipital parenchymal bleeding, as well as left frontal, temporal and parafalcine subdural hemorrhage with subfalcine herniation and midline shifting of > 1 cm to the right, hydrocephalus and signs of massively increased intracerebral pressure (Fig. 1a, b). Following the cCT scan, his clinical status rapidly deteriorated with both pupils wide and unresponsive to light. The patient was considered to have irreversible brain damage. As such, surgical decompression or osmotherapy was not an option and palliative care was initiated. The patient died a few hours later.

**Fig. 1:**
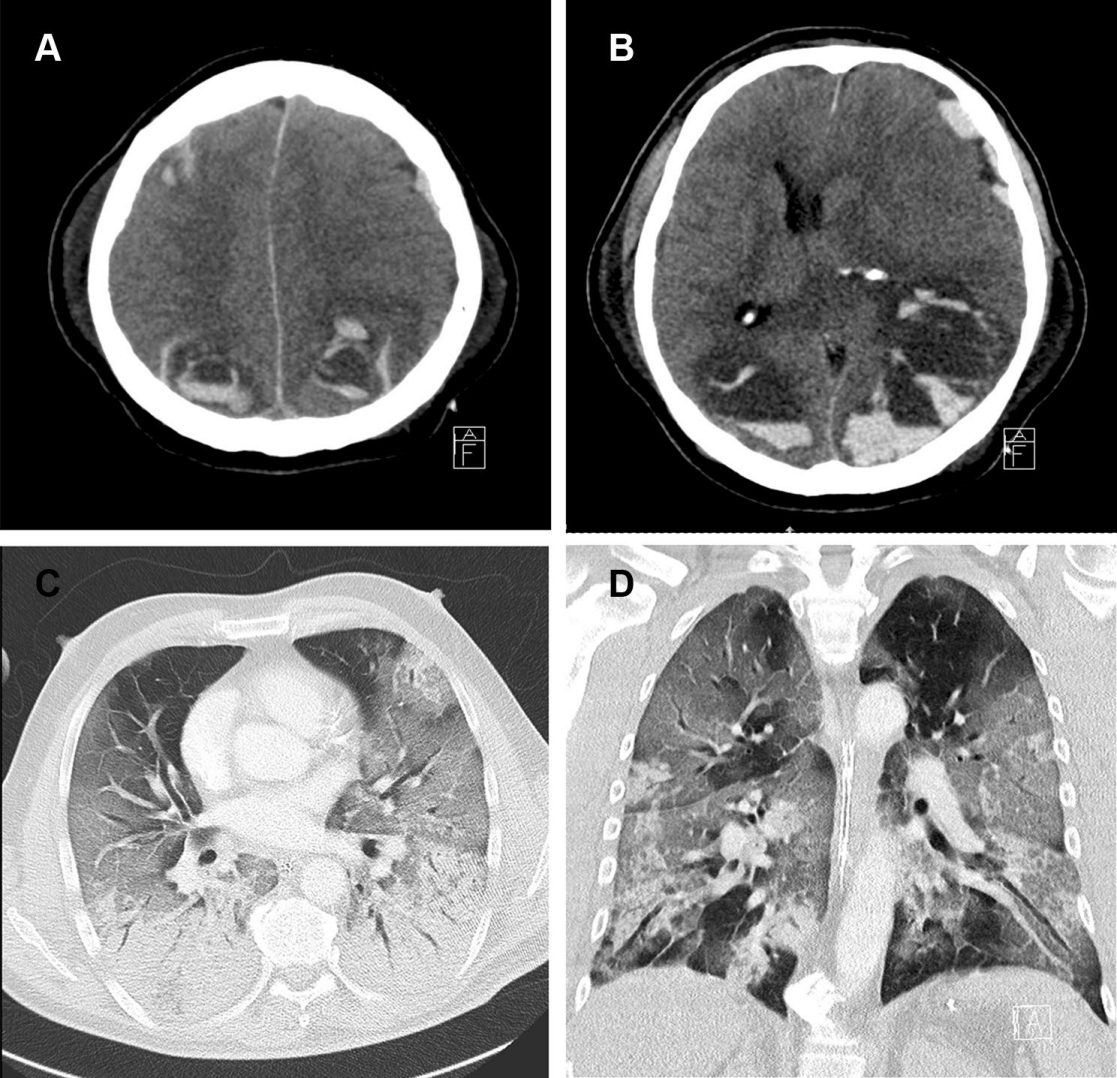
Imaging (CT) of the patient in the case report. **a** Bilateral hemorrhage in the parietooccipital parenchyma featuring fluid–fluid levels and left-hemispheric subdural hematoma. Considerable mass effect resulting in significant midline shift to the right as well as transtentorial and subfalcine herniation. **b** Right-sided subarachnoidal hemorrhage adjacent to small intraparenchymal bleeding. **c**, **d** Extensive ground glass opacities predominantly in the periphery of both lungs and dorsal air space consolidation

The aim of this case series is to review neurologic findings in high-risk intensive care patients suffering from moderate to severe COVID-19 induced ARDS. Furthermore, we will discuss the difficulty to decipher specific neurologic features of COVID-19 from epiphenomena of critical illness and the need for additional data on neurologic sequelae of COVID-19.

Reviewing all 38 COVID-19 patients admitted to our ICU between March 20th and May 27th 2020, we found neurological complications in nine (23.7%) cases (Table 1). Affected patients had a median age of 56 years (54–66), 66.7% were male and 33.3% female, median pO_2_/FiO_2_ ratio at admission was 157 mmHg (127–183). Seven patients had potentially life-threatening neurological events (18.4%), which in two cases occurred during veno-venous extracorporeal membrane oxygenation (ECMO) therapy. One patient died in consequence of the neurological complication (2.6%). Including the aforementioned case report, three patients suffered from intracerebral hemorrhage. A subarachnoid hemorrhage occurred in a tri-cytopenic patient with a history of stem cell transplant. Another patient showed multiple small intracerebral bleedings of septic-embolic etiology. Two patients had first-time generalized seizures, likely due to septic encephalitis and two suffered from transient paresis and aphasia. Ischemic insults were not confirmed.

**Table 1:** Neurological manifestations of critically ill COVID-19 patients (*n* = 38)

Demographics and ICU course	Patients (*n* = 38)
Female, *N* (%)	13 (34.2)
Male, *N* (%)	25 (65.8)
Age, years (median, IQR)	64.5 (19.5)
ICU admission	
Previous hospital stay, days (median, IQR)	3 (7)
PaO_2_/FiO_2_, mmHg (median, IQR)	143 (98.5)
SOFA score (median, IQR)	13 (4.5)
ICU course	
ICU stay, days (median, IQR)	20.5 (26.5)
Mechanical ventilation, days (median, IQR)	17.5 (17.3)
VvECMO, *N* (%)	15 (39.5)
Renal replacement therapy, *N* (%)	23 (52.6)
Highest SOFA score (median, IQR)	17 (6.5)
Minimal PaO_2_/FiO_2_, mmHg (median, IQR)	73 (36)
Neurological signs	
Patients with documented neurological manifestations, *N* (%)	9 (23.7)
Patients with fatal neurological manifestations, *N* (%)	1 (2.6)
Seizures, *N* (%)	2 (5.3)
Critical illness neuropathy, *N* (%)	2 (5.3)
Altered mental status, *N* (%)	5 (13.1)
Altered pupil motor response, *N* (%)	3 (7.9)
Difficulty speaking or understanding speech, *N* (%)	4 (10.5)
Aphasia, *N* (%)	2 (5.3)
Difficulty using fine motor skills, *N* (%)	1 (2.6)
Weakness, *N* (%)	4 (10.5)
Paresis, *N* (%)	2 (5.3)
Transient ischemic attack, *N* (%)	1 (2.6)
Brain imaging	
Cerebral white matter lesions, *N* (%)	1 (2.6)
Periventricular hypodensity, *N* (%)	1 (2.6)
Cerebral hemorrhage, *N* (%)	3 (7.9)
Neurological manifestations and vvECMO	
ECMO patients with neurological manifestations, *N* (%)	2 (5.3)
ECMO patients without neurological manifestations, *N* (%)	13 (34.2)
Patients with neurological manifestations and without ECMO, *N* (%)	7 (18.4)
Patients with neither neurological manifestations nor ECMO, *N* (%)	16 (42.1)
Outcome	
Survival upon ICU discharge, *N* (%)	28 (73.7)

*ICU* intensive care unit, *IQR* interquartile range, *N* number of patients, *PaO*_*2*_*/FiO*_*2*_ ratio of arterial oxygen partial pressure and fraction of inspired oxygen (Horovitz index), *SOFA* sequential organ failure assessment, *vvECMO* veno-venous extracorporeal membrane oxygenation

COVID-19 patients are threatened by ARDS and ICU treatment is required in approximately five percent of the cases [4, 9]. Stabilizing cardiopulmonary function is challenging; however, the success of intensive care and quality of life is often determined by the neurocognitive and neuromuscular function. Acute ischemic stroke, intracerebral bleeding and encephalitis have all been described in ARDS patients and long-term cognitive impairment is a major issue for 20% of ARDS survivors after 5 years [6]. Pathophysiologic changes contributing to neurological complications include hyperinflammation, blood–brain-barrier dysfunction, hypoperfusion and difficulties of mechanical ventilation [16]. Also, structural brain damage and neurological deterioration has been reported in 7% of non-COVID-19 patients following ECMO therapy [10].

In COVID-19, hypercoagulability and a high incidence of thromboembolism are additional problems [8, 14]. Intensivists are caught between Scylla and Charybdis: Hypercoagulability bears the risk of pulmonary embolism, stroke or cerebral infarction and prompts us to utilize therapeutic anticoagulation and low-dose ASA in all COVID-19 patients. On the other hand, a substantial risk of intracerebral hemorrhage needs to be considered.

It is unclear, whether the observed neurological events are sole epiphenomena of critical illness, or rather directly relate to the severe acute respiratory syndrome coronavirus 2 (SARS-CoV2) infection. In fact, data regarding the incidence of neurological complications in COVID-19 are scarce [2, 11] and the precise pathophysiology of SARS-CoV2 viral spread remains largely unknown. Corona viruses are neurotropic [3] and sudden loss of smell or taste can be early clinical signs [1]. Vascular alterations have been described in autopsies of COVID-19 patients and may also contribute to neurological manifestations and bleeding complications [12]. Few case series have described neurologic changes in COVID-19. Findings mainly include encephalopathy, encephalitis and changes in mental status, all of which might not be specific for COVID-19 [5, 7]. Nevertheless, early recognition of viral encephalitis, acute cerebrovascular disease and neural system damage seems critical, not only for short-term survival but long-term quality of life.

Despite a relatively high incidence of events compared to other ICU patients [13], our observations so far do not pinpoint towards COVID-19-specific neurological complications. This study highlights the importance of a careful use of full intense anticoagulation. We still believe, that the benefits of therapeutic anticoagulation outweigh the risk of severe intracerebral hemorrhage. However, many aspects of COVID-19 remain uncharted and the sheer number of patients requiring long-term neurologic care will provide a burden to all health care systems in the years to come.

## References

[1] Abalo-Lojo JM, Pouso-Diz JM, Gonzalez F (2020). Taste and smell dysfunction in COVID-19 patients. Ann Otol Rhinol Laryngol..

[2] Asadi-Pooya AA, Simani L (2020). Central nervous system manifestations of COVID-19: a systematic review. J Neurol Sci.

[3] Bohmwald K, Gálvez NMS, Ríos M, Kalergis AM (2018). Neurologic alterations due to respiratory virus infections. Front Cell Neurosci.

[4] Guan WJ, Ni ZY, Hu Y, Liang WH, Ou CQ, He JX, Liu L, Shan H, Lei CL, Hui DSC, Du B, Li LJ, Zeng G, Yuen KY, Chen RC, Tang CL, Wang T, Chen PY, Xiang J, Li SY, Wang JL, Liang ZJ, Peng YX, Wei L, Liu Y, Hu YH, Peng P, Wang JM, Liu JY, Chen Z, Li G, Zheng ZJ, Qiu SQ, Luo J, Ye CJ, Zhu SY, Zhong NS (2020). Clinical characteristics of coronavirus disease 2019 in China. N Engl J Med.

[5] Helms J, Kremer S, Merdji H, Clere-Jehl R, Schenck M, Kummerlen C, Collange O, Boulay C, Fafi-Kremer S, Ohana M, Anheim M, Meziani F (2020). Neurologic features in severe SARS-CoV-2 infection. N Engl J Med.

[6] Herridge MS, Moss M, Hough CL, Hopkins RO, Rice TW, Bienvenu OJ, Azoulay E (2016). Recovery and outcomes after the acute respiratory distress syndrome (ARDS) in patients and their family caregivers. Intensive Care Med.

[7] Holmes EA, O'Connor RC, Perry VH, Tracey I, Wessely S, Arseneault L, Ballard C, Christensen H, Cohen Silver R, Everall I, Ford T, John A, Kabir T, King K, Madan I, Michie S, Przybylski AK, Shafran R, Sweeney A, Worthman CM, Yardley L, Cowan K, Cope C, Hotopf M, Bullmore E (2020). Multidisciplinary research priorities for the COVID-19 pandemic: a call for action for mental health science. Lancet Psychiatry.

[8] Klok FA, Kruip MJHA, van der Meer NJM (2020). Incidence of thrombotic complications in critically ill ICU patients with COVID-19. Thromb Res..

[9] Livingston E, Bucher K (2020). Coronavirus disease 2019 (COVID-19) in Italy. JAMA.

[10] Lorusso R, Gelsomino S, Parise O, Di Mauro M, Barili F, Geskes G, Vizzardi E, Rycus PT, Muellenbach R, Mueller T, Pesenti A, Combes A, Peek G, Frenckner B, Di Nardo M, Swol J, Maessen J, Thiagarajan RR (2017). Neurologic injury in adults supported with veno-venous extracorporeal membrane oxygenation for respiratory failure: findings from the extracorporeal life support organization database. Crit Care Med.

[11] Mao L, Jin H, Wang M (2020). Neurologic manifestations of hospitalized patients with coronavirus disease 2019 in Wuhan, China. JAMA Neurol.

[12] Menter T, Haslbauer JD, Nienhold R (2020). Postmortem examination of COVID-19 patients reveals diffuse alveolar damage with severe capillary congestion and variegated findings in lungs and other organs suggesting vascular dysfunction. Histopathology.

[13] Ortega-Gutierrez S, Wolfe T, Pandya DJ, Szeder V, Lopez-Vicente M, Zaidat OO (2009). Neurologic complications in non-neurological intensive care units. Neurologist.

[14] Panigada M, Bottino N, Tagliabue P (2020). Hypercoagulability of COVID-19 patients in intensive care unit: a report of thromboelastography findings and other parameters of hemostasis. J Thromb Haemost..

[15] Ranieri VM, Rubenfeld GD, Thompson BT, Ferguson ND, Caldwell E, Fan E, Camporota L, Slutsky AS (2012). Acute respiratory distress syndrome: the Berlin Definition. JAMA.

[16] Sasannejad C, Ely EW, Lahiri S (2019). Long-term cognitive impairment after acute respiratory distress syndrome: a review of clinical impact and pathophysiological mechanisms. Critical Care (Lond, Engl).

[17] Singer M, Deutschman CS, Seymour CW, Shankar-Hari M, Annane D, Bauer M, Bellomo R, Bernard GR, Chiche JD, Coopersmith CM, Hotchkiss RS, Levy MM, Marshall JC, Martin GS, Opal SM, Rubenfeld GD, van der Poll T, Vincent JL, Angus DC (2016). The third international consensus definitions for sepsis and septic shock (sepsis-3). JAMA.

